# The Ability of North Island Robins to Discriminate between Humans Is Related to Their Behavioural Type

**DOI:** 10.1371/journal.pone.0064487

**Published:** 2013-05-20

**Authors:** Craig Barnett, Matt Salter, Clément Chevallier, Nicola Robertson, Otis Berard, Kevin C. Burns

**Affiliations:** 1 School of Biological Sciences, Victoria University of Wellington, Wellington, New Zealand; 2 Department of Life Science, Rikkyo University, Toshima-ku, Tokyo, Japan; 3 Centre d'Études Nordiques, Université du Québec à Rimouski, Rimouski, Canada; Monash University, Australia

## Abstract

Animals are able to learn to identify persistent threats to themselves and their offspring. For example, birds are able to quickly learn to discriminate between humans that have previously threatened their nests from humans with whom they have had no prior experience. However, no study has yet examined whether a bird's ability to discriminate between humans is related to the bird's underlying behavioural type. In this study, we examined whether there were differences among North Island (NI) robins (*Petroica longipes*), based on their underlying behavioural type, in their abilities to discriminate between familiar and novel human observers. Using a simple feeding experiment, we timed how long it took birds to attack a food item placed next to an observer on each of 7 days. On the eighth day, a different observer timed the birds. We found that birds could be split into two behaviour types based on their attack behaviour: fast attackers (latencies <20 sec) and slow attackers (latencies >20 secs). Interestingly, the fast birds did not increase their attack latency in response to the novel observer whereas the slow attackers did. This result, for the first time, demonstrates that a bird's ability to discriminate between humans can vary among birds based on their behavioural type.

## Introduction

Animals are selected to perceive risks posed by their environments and react to them adaptively (e.g., [1], [2]). Animals are able to quickly learn to recognise repeated or persistent threats (such as predators) in their environments and alter their behaviour to these threats in ways that minimise the risks to themselves or their offspring [3], [4]. One example of this type of learning is how birds respond to potential predators approaching their nests. Anecdotal accounts suggest that birds can quickly learn to discriminate between humans that have previously approached their nests from novel individuals [4]–[7]. Recently, birds were shown to leave the nest earlier and react more violently towards humans that had previously threatened their nest [8], [9]. Moreover, birds have also been shown to discriminate between humans in other dangerous situations such as when they have been captured and banded or after being chased by a specific person [10]–[12]. Heterospecific individual discrimination has also been found in many species of mammals [13]–[15], but whether it is a general avian cognitive trait is still a matter of debate [8], [10].

Heterospecific individual discrimination is a cognitive task because it requires the acquisition, retention, and recall of information [16] about the heterospecific individual, and the bird's interactions with these individuals. A widely used definition is that individual recognition occurs when receivers learn the unique cues of one individual and treat that individual differently from other individuals ([17], [18], but see [19]). Studies of heterospecific individual discrimination in birds have tended to assay individual responses to dangerous situations like humans approaching a nest or capturing and handling individuals which may be very stressful for birds and initiate an acute corticosterone (Cort) response in the birds [20], [21]. This Cort-response may heighten the bird's memory formation in these situations (e.g., [22]), which could enable birds to more readily learn dangerous stimuli. Moreover, given the adaptive significance of responding appropriately to highly dangerous situations, we might expect to find little variation in how individuals respond to risky situations [3]. However, there is growing evidence that there is much variation among individuals in their responses to risky situations such as predation threats.

Recently it has become clear that there can be much variation in individual's responses to predators both within populations (e.g., [23]) and among populations (e.g., [24]). However, few studies have examined whether individual birds within a population differ from one another in their ability to discriminate between individuals of another species. Within populations, different individuals manage risk differently from one another. For example, after being threatened by a predator, bolder individuals emerge from a refuge and resume foraging behaviour quickly whilst shy individuals take longer to emerge and resume foraging [23], [25]. Individual's stress responses are also related to their sex and behavioural character [26], [27]. These differences in individual's behavioural responses have recently become a focus for research into animal personality (see [28], [29] for reviews). However, this approach presents problems for traditional views of animal predator responses. This is because it has been thought that individuals within a population respond to specific predators in a uniform manner because this represented the optimal response to that predator [1]–[3]. When responses to a predator differ among individuals, this is traditionally explained in relation to extrinsic, social, or life history factors [1], or random variation around an optimal mean [28], [29]. Therefore, differences in responses among individuals to predators questioned the assumption that individuals within a population have a specific optimal behaviour in response to specific predators.

There has been an upsurge in research interest in the past decade focussed on individual behavioural differences under many guises (animal personality, temperament, coping styles, and behavioural syndromes all refer to similar behavioural phenomena) and for many behavioural traits [28]–[30]. For a behavioural trait to be considered a personality trait, individuals need to be consistent in how they express the behaviour (it is repeatable) and it should be related to the expression of other behavioural traits [29]. The propensity for individuals to take risks (boldness) is a behavioural trait that has been found to conform to this definition of an animal personality trait. It has been extensively studied and its expression found to be repeatable within individuals (e.g., [31]–[33] but see [34]) and it is also related to other behavioural traits such as aggressiveness (e.g., [35]) and exploratory behaviour (e.g., [31]). Personality traits in some cases have been shown to relate to differences in learning [36]–[40], which indicates that complex cognitive traits may also be related to personality in animals just as do other behavioural and life-history traits [28], [29]. Moreover, individuals with different behavioural types also have been shown to interact with their environments differently from one another. For example, fast exploring great tits (*Parus major*) and mice (*Mus musculus*) (which are also bolder) are faster to form routines than slow exploring animals [41], [42]. However, the relation between personality and complex cognitive tasks such as heterospecific individual discrimination remain largely unstudied [43].

In this study, we conducted a simple feeding experiment to determine whether North Island robins (*Petroica longipes*) (henceforth NI robins) were able to discriminate between a familiar and a novel observer. We measured the time it took subjects to eat a single mealworm (*Tenebrio molitor*) that was placed 1 m from the observer on each of eight days. On the first seven days, the human observer (who timed the birds latency to attack the mealworm) was the same person (the familiar observer) while on the eighth day; a novel observer timed the bird. We hypothesized that the ability of birds to discriminate between familiar and novel observers would be related to individual's behavioural types. Therefore, we predicted that birds that were fast to attack mealworms would not change their attack latency with the change the human observer whereas slow birds would. This is because birds that are fast to attack may also be faster to form routines, explore novel environments, and less detail orientated than slower individuals as has been found in other studies [41], [42].

## Materials and Methods

### Ethical considerations

The research was approved by the Victoria University of Wellington Animal Ethics Committee and was conducted in accordance with ASAB's Guidelines for the Treatment of Animals in Behavioural Research and Teaching.

### Study site, species, and protocol

The study was carried out from December 2010 until early March 2011 at Karori Wildlife Sanctuary (KWS), which is a 2.5 km^2^ reserve in central Wellington (41°18′ S, 174°44′ E). KWS is surrounded by a predator proof fence and has had all mammalian predators removed from within the reserve. Many rare and endangered endemic forest birds (of which NI robins are one species) have been reintroduced to this reserve. North Island robins are non-migratory medium-sized passerines. They forage predominantly on the ground for invertebrates in territories that they maintain throughout the year. We used birds along various walkways within KWS to collect our data. A significant minority of individuals were colour banded and we were able to distinguish other birds based on distinctive plumage patterns, marks (e.g., pox lesions), behavioural patterns, and the location of banded neighbours.

Prior to beginning experimental trials, birds were fed mealworms in order to map bird's territories and locate a focal point for data collection (normally for 2–3 days). We then left the birds for a minimum of 2 days before starting experimental trials. We ran experimental trials for each individual between 1000 and 1530 h (New Zealand daylight time [UTC +13 h]) over eight consecutive days. The closely related South Island robin (*Petroica australis*) can gain about 6% in body mass throughout the day although the greatest gains are early in the morning and the late afternoon [44]. Therefore, it is unlikely that differences in state explain the differences in the birds' performance in the trials between days or among individuals. Although we attempted to run trials in fine weather, this was not always possible, meaning that occasionally we collected data during inclement weather. However, this did not affect the birds' performance in the task once they were at the feeding point.

The experimental protocol was the same on each of the 8 days of the experiment, with slight modifications on day 8. The first 7 days, the same person (the familiar observer) wore a white laboratory coat over their clothing as they ran the trial. If the bird was not at the feeding point when the observer arrived, the observer waited until the bird came close to the observer. Most of the time, the bird approached the observer before they arrived at the focal point within their territory, but occasionally they had to wait 2–3 min until the bird arrived. Once the bird had approached to about 5 m of the observer, a beige 120 mm×120 mm ceramic bathroom tile was placed level on the ground about 1 m from the observer's foot. In the middle of the tile, a recently euthanized mealworm was placed. The observer then quickly (but smoothly) stood up and at the same time started a stopwatch and timed (to the nearest 0.1 sec) the latency for the bird to attack the prey (when its bill made contact with the mealworm). On the eighth day, the trial was run in the same way as the previous 7 days with two exceptions. These were that the person running the trial was different (the novel observer) and that they wore a light blue rather than a white coat over their clothing. After attacking and consuming the mealworm, the tile was collected and the observer walked from the bird's territory. The observer only removed the coat from over their clothes after they had left the bird's territory and could no longer see the focal bird. The colour of the coat was changed between days 7 and 8 because NI robins have a long evolutionary history without mammals and so are naïve to them. Although previous studies suggest that birds do not use clothing to distinguish between people (e.g., [8]), the change in coat colour provided a possible cue for the birds to use. Apart from the jacket, the other garments worn by familiar observers (e.g., trousers, footwear, and headwear) differed among days.

### Statistical analyses

**Figure 1:**
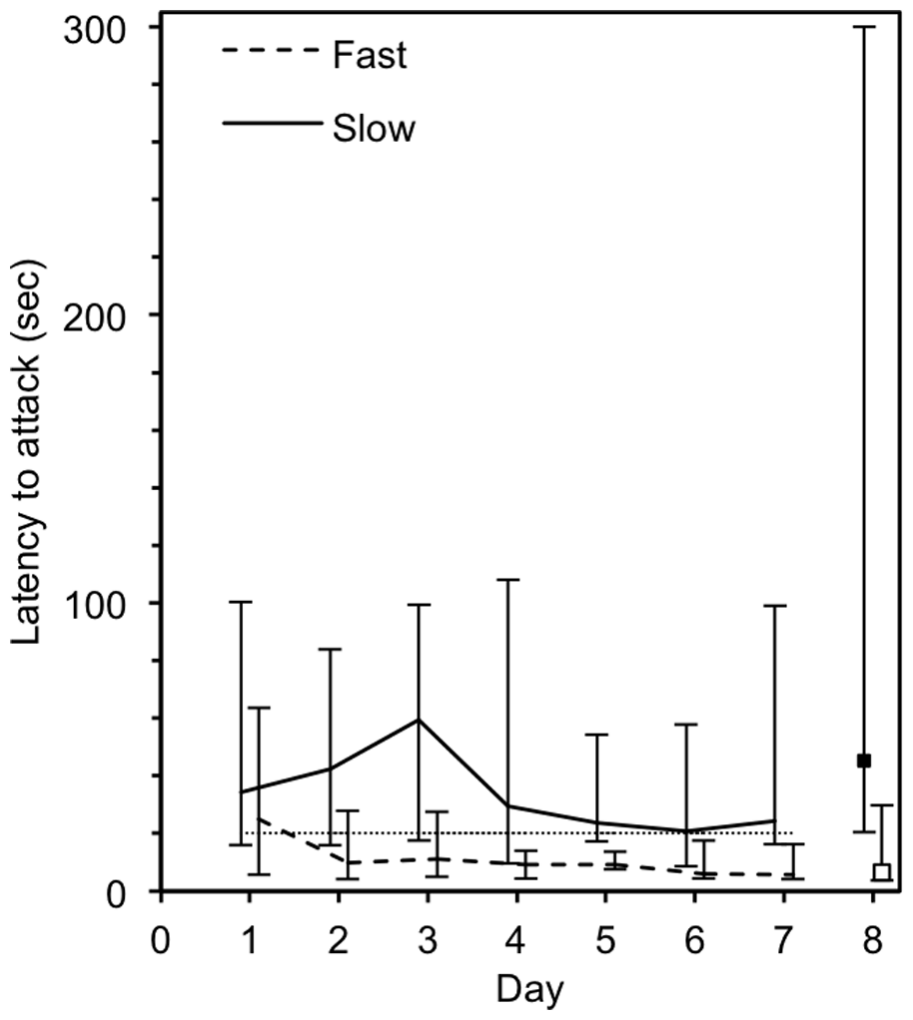
The median latencies over the course of the 8 days of the experiment for slow birds (solid line and filled symbol) and fast birds (hashed line and unfilled symbol). The bars represent the 10^th^ and 90^th^ percentiles. It is interesting to note that the latencies for the fast birds reach an asymptote on day 2 whereas slow birds reach their asymptote on day 3 of the experiment.

In order to confirm that the birds had two behavioural types we conducted two analyses on the data. First, we analysed the repeatability of the bird's latencies in order to confirm that their behaviour was consistent as this is a cornerstone of an animal personality trait [45]. To calculate repeatability (r), we used a well-established method [46]. We conducted a repeated-measures ANOVA on the log_10_ transformed latencies to calculate the within and between individual sum of squares. Second, we used the birds asymptotic attack latencies to arbitrarily sort them into two groups: fast and slow attackers. We classified the birds as being either fast or slow by averaging their latencies to attack from day 4 until day 7 (inclusive). We analysed the data from these four days to test whether the bird's attack latencies were asymptotic (i.e., they were stable with no significant differences among days). We used these four days' data because the bird's latencies to attack prey had reached an asymptote for both groups by day 4 (Fig. 1). We used this grouping variable in a discriminant function analysis and the log_10_ transformed data from day 1 through to day 7 as well as the difference in the latency between day 7 and day 8 to confirm that our grouping variable was a reliable estimator of individual behaviour.

In order to analyse the difference in attack latencies between day 7 (familiar observer) and day 8 (novel observer), we subtracted the attack latency on day 7 from the attack latency on day 8. We then ran a generalised linear model on the difference in latency between day 7 and day 8 as the dependent variable and bird's sex and their behaviour group (i.e., fast or slow) as independent variables as well as the interaction between these two variables. We used a Gaussian distribution and an identity-link function and used the “glm” command in the glm package of R [47]. Age was not included in the analysis because all birds were adults and their exact ages were unknown.

## Results

### Repeatability

The latency to attack prey was significantly repeatable in individual birds (r = 0.5406, *F*_25,150_ = 9.2376, *P*<0.0001). This means that a high proportion (repeatability [r] = 0.54) of the variance in the attack latencies was due to differences among individuals and that the variance within individual behaviour was low and therefore repeatable.

### Group membership

A threshold of 20 sec was a reliable estimator of whether individuals were fast or slow at attacking prey. Of the 25 individuals from which we were able to collect complete data sets, 25 individuals (100%) were correctly assigned to their respective groups (14 individuals fast, 11 individuals slow). Results from the discriminant function analysis revealed 1 function with an eigenvalue of 4.665 and explaining 100% of the variance (χ^2^ = 32.95, df = 8, *P*<0.001). Figure 1 also suggests that the fast birds may have been quicker to reach an asymptote for their attack latencies, reached on day 2, compared with the slow birds who seemed to reach an asymptote on day 4.

### Effect of novel observer on attack speed

**Figure 2:**
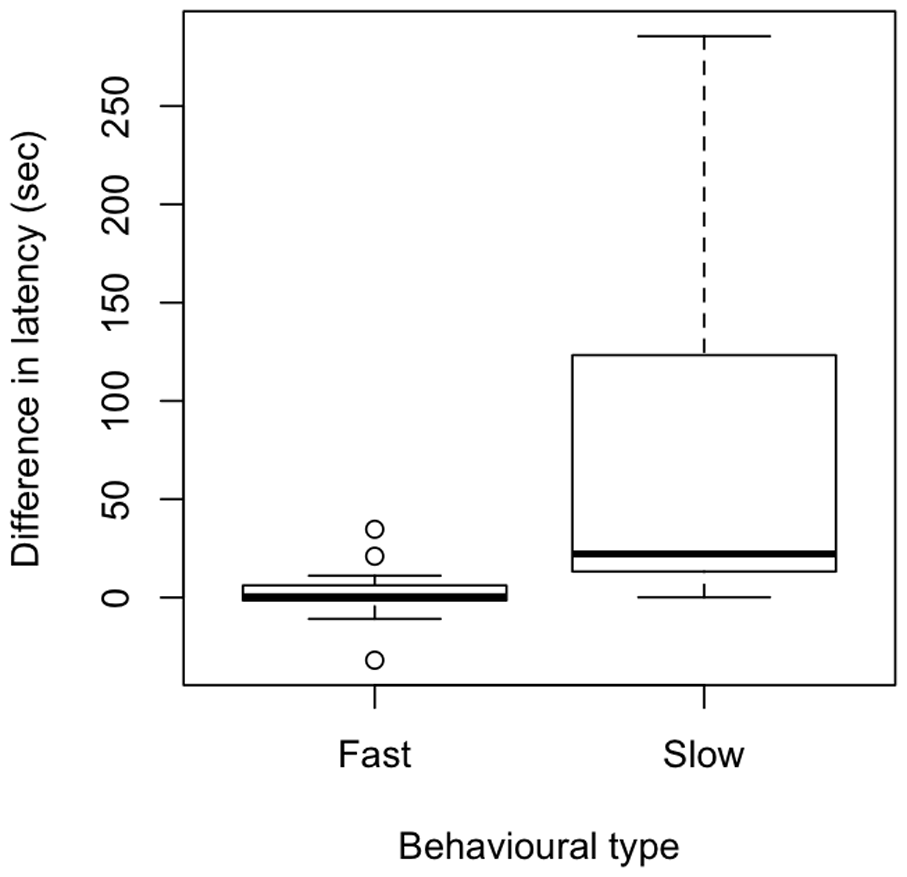
The median difference in the latency to attack prey for the novel person (day 8) minus latency for the last day familiar person (day 7) for fast and slow birds. Positive values indicate that birds took longer to attack prey when the novel observer was timing the birds compared with a familiar observer. The box indicates the first and third quartiles whilst the bars indicate the 10^th^ and 90^th^ percentiles.

We found that there was a significant difference in how birds with the two behavioural types responded to the novel observer (χ^2^ = 6.358, df = 1, P = 0.012, Fig. 2). Birds that had been fast to attack prey between days 4–7 did not increase their attack latencies when timed by novel observers. However, individuals that had been slower to attack prey between days 4–7 increased their attack latencies when they were timed by the novel observer.

## Discussion

We have shown that there is a difference between behavioural types of NI robins in their responses to familiar and novel human observers and that this difference is related to a difference in the bird's behavioural type. Birds that discriminated between familiar and novel observers were slower to attack mealworms during the first 7 days of the experiment. Conversely, birds that did not discriminate between familiar and novel observers were faster to attack prey. Previous studies have shown that birds [8]–[12] and mammals [13]–[15] are able to identify individual humans, which is a form of heterospecific individual discrimination. However, this is the first study to show that individual differences in this ability might be related to differences in behavioural type.

Our results are consistent with previous studies that examined how different behavioural types respond to change and novelty. For example, figure 1 shows that the fast birds reached an asymptote on day 2 whereas slow birds did not reach an asymptote until day 4, which suggests that birds with the fast behavioural type are faster to form routines than the birds in the slow group. Although we did not assess individual exploration, boldness has been found to be positively correlated with exploration. Therefore, our results might be consistent with findings that birds that are faster at exploring a novel environment are also quicker to form routines [38]. These earlier studies show that in great tits, boldness and other behavioural traits are related to one another, and that bolder individuals are quicker to form routines.

Recent work [10] on American crows (*Corvus brachyryhchos*), shows that some individuals were inaccurate at discriminating between people that had previously been dangerous or neutral and scolded both types of people. The authors suggested that these individuals might have been poor discriminators although they had no data on these individuals to support this possibility. However, it is an enticing possibility that crows' abilities to discriminate among humans is also related to differences in their behavioural types.

It is difficult to know if the fast birds did not perceive a difference between the familiar and the novel observers or if they did notice a difference, but it did not affect the bird's perception of risk. There are two possible explanations for the difference in discrimination between behavioural types. First, the two behavioural types may have devoted different amounts of attention to the task, which could result in differences in the amount of information that they acquire, process, and store [48]. The fast birds might pay less attention to the task and so learn fewer details of the environment associated with the experiment. Second, the fast and the slow birds might have different endocrine profiles. Slow birds might produce more corticosterone in relation to the risk associated with approaching a person [20], [21], which may enhance memory formation (e.g., [22]). Therefore, slow birds might have formed better memories of the daily task and so have a better template on which to compare the task among days. However, these two explanations are not mutually exclusive and might both be responsible for the observed results.

Previous studies of heterospecific individual discrimination have predominantly been conducted in urban contexts. There have been two hypotheses proposed to explain why urban species might be good at discriminating between humans. The first hypothesis is based on the fact that birds living in urban environments have high nesting success. Therefore, there could be an association between the perceptive abilities of birds and that rate at which they learn about sources of danger (such as predators) in their environment [9]. Therefore, bird species that are very perceptive and quick learners (and have larger relative brain sizes [49], [50]) might be predisposed to success in urban environments. The second hypothesis supposes that species that are exposed to humans regularly in urban or agricultural environments are exposed to humans at higher rates and so have been in situations that provide a greater number of experiences on which to base discrimination of humans [10]. North Island robins are an anomaly because they are capable of complex cognitive tasks (e.g., [51], [52]), but have (so far) failed to adapt to urban environments. Their failure to adapt to urban environments is probably because of life history constraints and a lack of behavioural defences against mammalian predators rather than a lack of cognitive ability or a lack of exposure to humans [53], [54]. Although it may be surprising that small passerines are able to discriminate between humans, it is probable that many avian taxa, under some circumstances, need to remember salient information about their environment. For example, many charadriidae species are able to discriminate between subtly different shadow features related to different predator species [55], [56]. Therefore, the cognitive mechanisms to make the types of discriminations required for heterospecific individual discrimination may be widespread among birds so long as these problems are presented to them in contextually relevant ways [8], [12]. We encourage further research to ascertain if such discrimination abilities are a general avian cognitive trait.

Behavioural traits that are part of an animal's behavioural type (or personality) should be consistent within the same individual when tested repeatedly [44]. This practically means that animals ought to maintain their relative rank within a population with regards to the behaviour being tested. The birds' responses during our trials were highly repeatable which would tend to suggest that individual's latencies to attack prey were consistent. Evidence is growing that repeatability declines with increased time between testing [45], [57], [58]. However, previous studies have used intervals as short as those used in this study [58]. It is also likely that our measure of repeatability was related to learning and it is difficult to disentangle repeatability and learning [59]. However, the focus of this study was to examine if there was variation among birds in their abilities to discriminate between heterospecific individuals. Therefore, it would be difficult to design an experiment to test for repeatability of heterospecific individual discrimination that did not involve learning.

In conclusion, we have found that different behavioural types within a population respond differently to being observed by a novel human. Whilst individuals of many bird species [8]–[12] and other taxa [12]–[14] have been shown to recognise individual human individuals, this is the first demonstration that different behavioural types might perform this task differently. Therefore, our data are consistent with earlier studies that have shown that some behavioural traits (such as exploration and boldness) are related to routine formation and level of neophobia [41], [42]. Moreover, these results emphasise that individual differences in behaviour types may need to be considered when studying complex cognitive traits. We suggest that future studies should focus on examining the relation between animal's behavioural types we uncovered in this study and performance in other cognitive tasks such as caching [51] in order to uncover whether risk-taking behaviour is related to more general cognitive performance. We also encourage further research to uncover whether heterospecific individual discrimination is a general avian cognitive trait.
